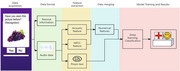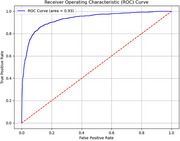# A Multimodal Fusion Framework for Early Detection of Cognitive Impairment in Chinese Speakers Using Pinyin Sequences and Acoustic Features

**DOI:** 10.1002/alz70856_100110

**Published:** 2025-12-24

**Authors:** Liangwei Tao, Zhixing Zhou, Huanhuan Xia, Quan Chen, Yiming Li

**Affiliations:** ^1^ University of Shanghai for Science and Technology, Shanghai, China; ^2^ Shanghai Bestcovered Limited, Shanghai, China; ^3^ Shanghai University of Medicine & Health Sciences, Shanghai, China

## Abstract

**Background:**

Alzheimer's disease (AD) is a leading cause of dementia, and traditional diagnostic methods like cerebrospinal fluid testing and PET imaging are invasive, costly, and limit early detection. Language biomarker analysis offers a non‐invasive, efficient alternative to detect cognitive impairments through speech. However, in Chinese, the presence of homophones often leads to transcription errors, which may reduce model accuracy. Converting text to Pinyin sequences can minimize ambiguity, enhancing detection. This study proposes a novel speech‐based method to improve cognitive impairment detection accuracy with greater efficiency.

**Method:**

This study utilized a systematic approach to differentiate individuals with cognitive impairment from healthy controls (HC). With approval from the hospital ethics committee, data from 300 participants in the China Preclinical Alzheimer's Disease Study (C‐PAS) cohort were extracted. Audio data were transcribed using iFLYTEK's speech recognition tool and converted into Pinyin sequences. Acoustic features, such as pause frequency and silent time, were extracted using OpenSMILE, and MFCC features were also incorporated. These features, along with demographic variables, formed comprehensive digital signatures for model training. To address the small sample size, data augmentation techniques such as introducing noise to numerical features and simulating word omissions, repetitions, and replacements in Pinyin sequences were applied. A Bi‐directional LSTM model, known for capturing context and semantic relevance, was employed to fuse Pinyin sequences with numerical features and optimize classification performance.

**Result:**

The proposed method achieved an accuracy of 93.80% and an Area Under the Curve (AUC) of 0.93, demonstrating its superior performance compared to models trained solely on acoustic features or cognitive test scores. Ablation experiments revealed that combining pinyin sequences with acoustic features significantly enhanced model performance, emphasizing the importance of integrating both linguistic and acoustic data for detecting Alzheimer's disease in Chinese.

**Conclusion:**

This study demonstrates the feasibility and effectiveness of integrating Pinyin sequences and acoustic features for non‐invasive Alzheimer's detection in Chinese. These findings providing a practical tool for early screening and paves the way for larger‐scale studies and potential clinical application.